# Results of Pancreatic Blood Shunting into the Systemic Blood Flow in Insulin-Dependent Diabetics

**DOI:** 10.1155/1996/41904

**Published:** 1996

**Authors:** E. I. Galperin, T. G. Diuzheva, P. F. Petrovsky, A. Yu. Chevokin, K. V. Dokuchayev, S. E. Rabinovich, E. P. Gitel, N. F. Kuzovlev, L. V. Platonov

**Affiliations:** 1 Department of Hepatic and Metabolic Surgery I.M. Sechenov Medical Academy Moscow Russia; 2 39 Bolshaya Gruzinskays Street ap. 45 Moscow 123056 Russia

## Abstract

A new surgical method of treating patients with unstable insulin-dependent diabetes (IDD) has been developed-that of surgically shunting pancreatic blood into the systemic blood flow with the purpose of creating a more optimal interaction of subcutaneously administered insulin and pancreas-secreted glucagon.

The long term results of the operation depend on the patency of a splenorenal anastomosis. This has been studied by following up 137 patients over periods from half a year to three years. Anastomotic patency was determined by renal and splenic venography and celiacy arteriography, which revealed a patent anastomosis in 114 patients, and an obliterated one in 23.

Patients with patent anastomoses showed a lowering of glycosylated hemoglobin (HbA_lc_) from 13.3±0.03% to 9.3±0.6%, p<0.05, a decrease of the injected insulin dose from 0.97±0.04 to 0.72±0.03 U/ kg, p<0.05, disappearance or considerable abatement of pain in the lower extremities, and of hypoglycemia. Improvement of clinical status was accompanied by an increase of glucagon in the systemic blood stream from 60.8±10.1 to 91.5±9.4 pg/ml, p<0.05, a rise of tissue oxygen pressure, PO_2_, from 49.2±2.4 to 58.1±1.9 mm Hg, p<0.05. In patients with oblivious anastomoses postoperative HbA_lc_ levels did not change from preoperative values: 12.9±0.4% and 12.8±0.7%, p<0.05, respectively; the insulin dose remained the same-0.91 ±0.07 U/kg and 0.85±0.07 U/kg, p<0.05, no rise of the systemic blood glucagon content was noted, and former complaints continued.

The suggested method is not an alternative for insulin therapy, but considerably enhances its potential.


*HPB Surgery*, 1996, Vol.9, pp.191–197
Reprints available directly from the publisher
Photocopying permitted by license only

© 1996 OPA (Overseas Publishers Association)
Amsterdam B.V. Published in The Netherlands
by Harwood Academic Publishers GmbH
Printed in Malaysia

### Results of Pancreatic Blood Shunting into the Systemic Blood Flow in Insulin-Dependent Diabetics

E.I. GALPERIN,* T.G. DIUZHEVA, P.F. PETROVSKY, A. Yu. CHEVOKIN, K.V. DOKUCHAYEV, S.E. RABINOVICH, E.P. GITEL, N.F. KUZOVLEV and L.V. PLATONOVA

Department of Hepatic and Metabolic Surgery, I.M. Sechenov Medical Academy, Moscow, Russia.

*(Received 24 February 1994)*

A new surgical method of treating patients with unstable insulin-dependent diabetes (IDD) has been developed–that of surgically shunting pancreatic blood into the systemic blood flow with the purpose of creating a more optimal interaction of subcutaneously administered insulin and pancreas-secreted glucagon.

The long term results of the operation depend on the patency of a splenorenal anastomosis. This has been studied by following up 137 patients over periods from half a year to three years. Anastomotic patency was determined by renal and splenic venography and celiacy arteriography, which revealed a patent anastomosis in 114 patients, and an obliterated one in 23.

Patients with patent anastomoses showed a lowering of glycosylated hemoglobin ($HbA_{1c}$) from 13.3±0.03% to 9.3±0.6%, $p < 0.05$, a decrease of the injected insulin dose from 0.97±0.04 to 0.72±0.03 U/kg, $p < 0.05$, disappearance or considerable abatement of pain in the lower extremities, and of hypoglycemia. Improvement of clinical status was accompanied by an increase of glucagon in the systemic blood stream from 60.8±10.1 to 91.5±9.4 pg/ml, $p < 0.05$, a rise of tissue oxygen pressure, $pO_2$, from 49.2±2.4 to 58.1±1.9 mm Hg, $p < 0.05$. In patients with oblivious anastomoses postoperative $HbA_{1c}$ levels did not change from preoperative values: 12.9±0.4% and 12.8±0.7%, $p < 0.05$, respectively; the insulin dose remained the same –0.91±0.07 U/kg and 0.85±0.07 U/kg, $p < 0.05$, no rise of the systemic blood glucagon content was noted, and former complaints continued.

The suggested method is not an alternative for insulin therapy, but considerably enhances its potential.

KEY WORDS: Insulin-dependent diabetes liver pancreas distal venous splenorenal anastomosis

### INTRODUCTION

It is common knowledge that the basis of the pathogenesis of insulin-dependent diabetes (IDD) is insufficiency of insulin. The role of insulin antagonists, glucagon among them, in disturbances of glucose metabolism still remains unclear. Some reports indicate that glucagon enhances glucose production by the liver, and that its secretion in the pancreas of IDD patients increases [1-4]. Moreover, it has been demonstrated that also enhanced in IDD patients is the function of the contrinsular hormones of the adrenal glands [1].

Glucose homeostasis is maintained by the production of glucose in the liver and its utilization by peripheral tissues; it is controlled by the interaction of insulin and anti-insulin hormones. Exogenous insulin is injected into IDD patients subcutaneously, with only part of it reaching the liver where-as all the endogenously secreted glucagon enters the liver via the splenic and portal veins. The lack of correlation of the injected insulin with endogenous glucagon in the tissues may be one of the causes of the insufficient effect of insulin therapy.

Experimental research we carried out earlier in two diabetes mellitus models in dogs (subtotal resection of the pancreas and alloxan administration) demon-

*Correspondence to*: *Professor E.I. Galperin, 39 Bolshaya Gruzinskays Street, ap. 45, 123056 Moscow, Russia. Tel. (095) 118–82-38

strated that deflection of venous blood from the pancreas into the systemic blood flow, bypassing the liver, results in the lowering of glycemia and reduction of the level of triglycerides[5–7].

Since 1986 we have performed the operation of placing a distal venous splenorenal anastomosis in 409 IDD patients. The indication for surgery was a form of the disease resistant to insulin treatment, with an unstable course and rapid progression of microangiopathy. The operation allows shunting of the venous blood flowing from the pancreas into the systemic blood flow, which should reduce the effect of glucagon on the liver, improve the correlation between injected insulin and endogenous glucagon both in the liver and in peripheral tissues. Long term results of surgery over periods from 2 to 5 years were followed up in 207 persons. Stabilization of the course of diabetes and the development of angiopathy, accompanied by the disappearance or abatement of complaints of fatigue and pain in the lower extremities, occurred in 75 percent of cases.

The purpose of this investigation was to determine how remote results of surgery depend on the patency of the splenorenal anastomosis.

### MATERIALS AND METHODS

Angiographic examinations of 137 randomized IDD patients were carried out a considerable time after surgery. In order to demonstrate that the results depended on anastomotic patency rather than anything else, apart from the randomized group, patients were taken at various postoperative periods: 74 at 6 months, 54 at a year, 8 at two years, and 4 at three years after the operation. This corresponded to the sequence of their readmission for follow-up examinations. Patients who declined to undergo angiography were excluded from the study.

The age of the patients varied from 16 to 52 years (median age – 29.5±0.4 years), duration of disease from 1.5 to 27 years (median – 10.5±0.3 yrs). A labile course of DM was observed preoperatively in 72 of the patients, 31 of them had a history of hypoglycemic coma, 23 of hyperglycemic coma, 18 a combination of hypo- and hyperglycemic comas. The median dose of exogenous insulin was 0.9±0.02 U/kg, median daily glycemia – 12.2±0.3 mM/liter, the level of glycosylated hemoglobin – 13.3±0.3%.

### *Surgical Techniques*

Laparotomy was performed. The splenic and renal veins were approached through the lesser sac. The parietal peritonium was dissected along the lower edge of the pancreas to mobilize the splenic vein at a distance of 3–5 cm from the root of the portal vein. The small pancreatic branchlets were carefully ligated and dissected. Another incision of the parietal peritonium was made in the projection of the left renal vein, which was mobilized at a distance of 4–5 cm. After placing clamps the splenic vein was ligated and dissected at the point where it formed the portal vein, an aperture was formed in the renal vein and an end-to-side splenorenal anastomosis was created with a continuous vascular suture, using 5/0–7/0 Prolene and catgut.

### *Angiographic Techniques*

Angiographic examinations were made with a GEM unit from Thomson Benelux Co., and using a Mark IV system from Medrad Co. for the automatic administration of the Verographin 60% contrast medium. Examination was started with renal venography by the catheterization of the left renal vein using the Seldinger technique, using a 'pig-tail' catheter. When possible, selective splenic venography was performed if the splenic vein was amenable to catheterization via the region of the splenorenal anastomosis. A catheter of original design was used for this purpose. In cases when the above techniques failed to reveal the splenic vein, arteriography was carried out, for which a hook-type catheter was used. In addition to the arterial phase, the distribution of the contrast medium into the reverse venous phase was studied. In 14 patients presenting with patent anastomoses, both renal venography and arteriography were performed in order to verify the anurons of radiograph interpretations in patients with patent and obstructed anastomoses. All the examinations were additionally videorecorded.

### *Biochemical and Hormonal Investigations*

Blood glucose levels were determined by the ortho-toluidine technique. Blood was collected by fingertip puncture five times within 24 hours. Glycolysated hemoglobin ($HbA_{1c}$) was determined by the method of affinity chromatography[8] with the use of borphenyl agarose as sorbent.

The hormonal profile was assessed by the level of c-peptide, glucagon and cortisol. Radioimmune assay kits from Byk-Sangtech (FRG), Serono (Switzerland), and Amersham (U.K.) companies were used.

Kidney function was assessed by the rate of glomerular filtration, determined by 24-hour endogenous creatinine clearance.

*Evaluation of Tissue Oxygenation*

Tissue oxygen balance was determined by the gas content of the blood with the aid of an apparatus from Corning Co. (U.K.). Bood samples for $pO_2$ and $pCO_2$ were drawn from the artery and vein of the forearm. Percutaneous measurements were taken with the aid of a TM-220 oxymonitor from Radiometer Co. (Denmark), fitted with heating electrodes (43°C). The electrodes were secured on the lower third of the shin.

*Evaluation of the Vegetative Nervous System*

The state of diabetic autonomic neuropathy was determined from the degree of respiratory arrhythmia during quiet ($G_1$) and forced ($G_2$) respiration, by the 30/15 index [9]. The degree of respiratory arrhythmia was assessed by the mean square variational deviation in the duration of cardiac intervals during quiet and forced respiration (3 deep 5-second-long inhalations and exhalations) in milliseconds. The 30/15 index was determined by correlation of the length of the thirtieth cardiac interval to that of the fifteenth cardiac interval after the patient changed from the horizontal to the vertical position. The pulse rate was determined from the mean cardiac interval length at quiet breathing.

*Statistical Processing*

The data obtained were processed on an IBM PC/AT computer, using a Statographics statistical package, USA.

### RESULTS

*1. Immediate Results of Surgery*

All the 137 patients withstood the operation well, and were duly discharged from the clinic. Complications occurred in 11 cases: 4 had an exacerbation of chronic pyelonephritis, 5–focal pneumonia, and 2–fistulae in the region of the surgical suture line.

*2. Results of Angiography*

Renal venography was performed in 79 patients, in 26 of whom there was contrast visualization of the splenic vein, indicating patency of the splenorenal anastomosis (Figure 1). In 14 patients renal venography was combined with arteriography, both techniques revealing the filling of the splenic vein. There was no filling of the splenic vein in 53 patients, so all of them underwent arteriography. In the reverse venous phase 30 patients showed an image of the splenic vein to the level of the catheter placed in the left renal vein (Figure 2), indicating patency of the anastomosis. In the other 23 patients contrast filling of the splenic vein either never occurred, or was detected as a short length from the pancreas with a gap between this portion and the catheter placed in the left renal vein (Figure 3). The anastomosis in these 23 patients was held to be obstructed. In 19 patients arteriography was performed as the only method; constrast filling of the splenic vein was observed. Selective splenic venography was performed in 39 patients, showing anastomotic patency (Figure 4). Thus, angiography found a patent anastomosis in 114 persons (Group 1) and an obliterated anastomosis in 23 persons (Group 2). Before surgery the patients in the two groups did not differ in duration of the disease (10.3±0.73 and 9,6±2.1 years respectively, $p > 0.05$), age (29.5±1.04 and 26.8±2.1 years), exogenous insulin dose (0.92±0.03 and 0.87±0.05 U/kg, $p > 0.05$) or mean daily glycemia level (11.9±2.7 and 13.2±1.1 mM/liter, $p > 0.05$).

*3. Exogenous Insulin Dose*

In patients with patent anastomoses the insulin dose was postoperatively reduced from 0.97±0.04 to 0.72±0.03 U/kg, $p < 0.05$. In patients with blocked anastomoses the dose did not significantly change: 0.91±0.07 U/kg and 0.85±0.07 U/kg. $p > 0.05$. The insulin preparation and pattern of administration did not change in these patients.

*4. Changes in Glycolysated Hemoglobin*

Before surgery the patients in Groups 1 and 2 hardly differed in $HbA_{1c}$ levels: 13.3±0.3 and 12.9±0.4% respectively, $p > 0.05$. After the operation patients with a patent anastomosis showed a drop in the $HbA_{1c}$ level to 9.3±0.6%, $p < 0.05$; patients with obstructed anastomosis showed no change: 12.8±0.7%. $p > 0.05$.

*5. Hormonal Study Findings*

These studies were carried out in 31 patients with patent anastomoses and 11 with impassable ones. Before surgery the c-peptide levels in these patients prac-

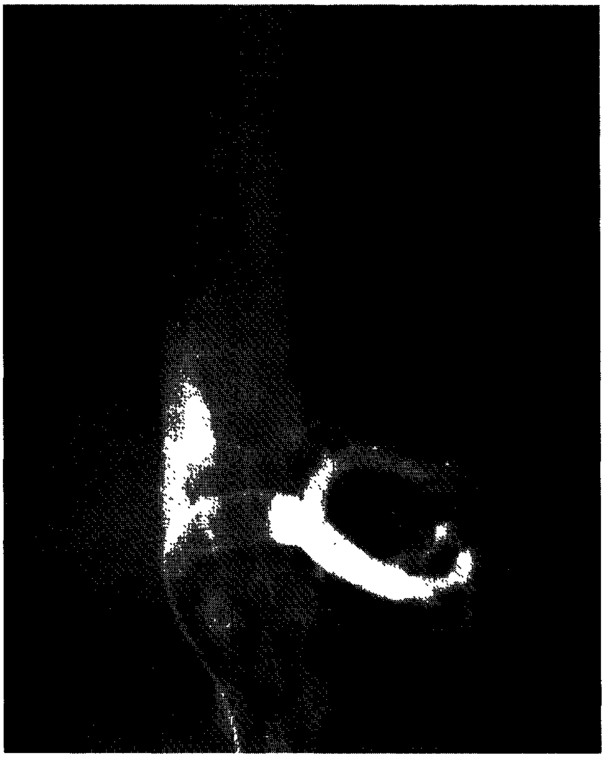

**Figure 1** Renal venogram of patent splenorenal anastomosis.

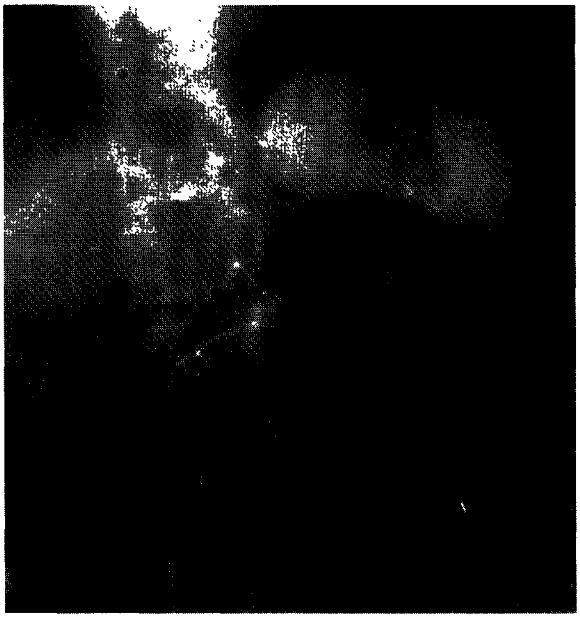

**Figure 2** Arteriogram. Reverse venous phase with patent splenorenal anastomosis.

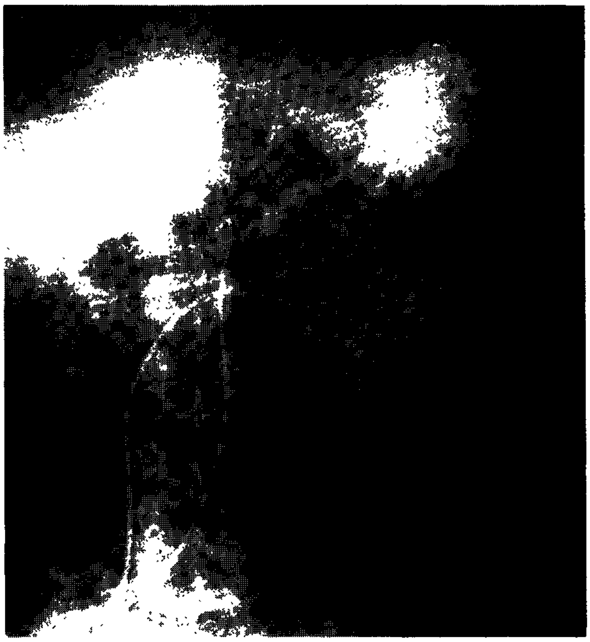

**Figure 3** Arteriogram. Reverse venous phase with impassable anastomosis (see text).

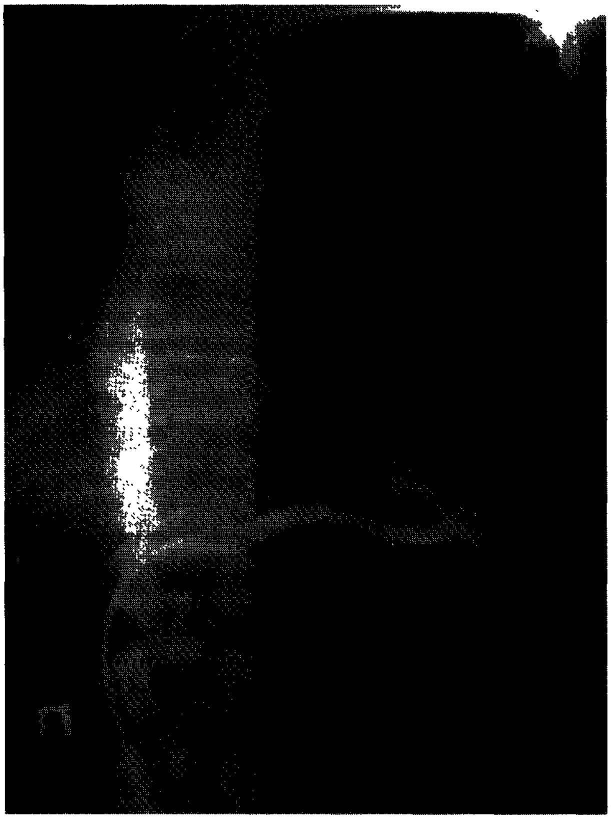

**Figure 4** Selective splenic venogram.

tically did not differ, being 0.45±0.17 and 0.33±0.16 ng/ml respectively, $p > 0.05$. After surgery the c-peptide values in patients with patent anastomoses increased to 0.84±0.18 ng/ml, $p < 0.05$, while in those with impassable anastomoses they did not change: 0.26±0.08 ng/ml, $p > 0.05$.

Nor did the two groups differ in preoperative glucagon content. 60.8±10.1 pg/ml and 77.8±10.0 pg/ml, $p > 0.05$. After surgery the glucagon content in patients with patent anastomoses increased to 91.5±9.4 pg/ml, $p < 0.05$, in those with impassable anastomoses there was no change: 69.6±9.0 pg/ml, $p > 0.05$.

The dynamics of anti-insulin hormones (glucagon and cortisol) was examined pre- and postoperatively in 9 patients (Table 1). The splenorenal anastomosis was passable in all of them. After surgery the glucagon level in the systemic blood flow increased in all the patients, and this was accompanied by a drop of the cortisol content in 8 persons.

### *6. Filtering Capacity of the Kidneys*

The glomerular filtration rate (GFR) was examined in 96 patients. They were divided into 3 groups according to preoperative GFR findings: 41 patients had a diminished GFR at 64.9±1.6 ml/min, 34 patients had normal GFR at 97.7±2.1 ml/min, and in 21 it was increased to 164.0±6.1 ml/min. In patients with a patent anastomosis low filtration rates rose to 90.0±7.3 ml/min, $p < 0.05$ (29 persons), normal filtration did not change–102.6±5.8 ml/min (30 persons), elevated GFR tended to normalization –143.3±13.8 ml/min, $p > 0.05$ (21 persons). In patients with an impassable anastomosis and low GFR, it did not rise – 72.7±7.8 ml/min. $p > 0.05$ (12 persons), those with normalfiltrations showed a drop to 73.3±3.6 ml/min, $p < 0.05$ (4 persons).

### *7. Tissue Oxygen Balance*

The state of tissue oxygenation was studied in 25 patients. Preoperative epicutaneous measurement found $pO_2$ equal to 49.2±2.4 mm Hg, $pCO_2$ = 44.4±0.98 mm Hg. After surgery the $pO_2$ of 21 patients with patent anastomoses increased to 58.1±1.93 mm Hg, $p < 0.05$. The $pCO_2$ did not change –41.7±1.2 mm Hg. In patients with impassable anastomoses the epicutaneously measured $pO_2$ was low –39.6±2.7 mm Hg, $p < 0.05$; the $pCO_2$ did not differ from the preoperative level. In patients with a patent anastomosis (n = 21) the arterial blood $pO_2$ was 93.6±5.8 mm Hg, in venous blood it was 22.8±2.7 mm Hg; arterial blood $pCO_2$ was 26.7±1.9 mm Hg, in venous blood it was 41.3±3.3 mm Hg. In patients with an obstructed anastomosis (n = 4) arterial blood $pO_2$ was 97.5±2.1 mm Hg, in venous blood it was 30.2±1.5 mm Hg; arterial blood $pCO_2$ was 36.5±0.7 mm Hg, in venous blood it was 41.8±3.2 mm Hg.

### *8. Vegetative Innervation of the Heart*

This was studied in 26 patients before and after surgery. The splenorenal anastomosis was patent in 22. The $G_1$ index increased from 25.4±2.9 msec by 10.5±3.4 msec, $p < 0.01$; $G_2$–from 62.8±8.0 msec by 21.9±6.9 msec, $p < 0.01$; the 30/15 index - from 1.065±0.02 by 0.092±0.035, $p < 0.05$. Here the pulse rate decreased from 79.9±3.0 by 5.8±2.2 pulsations per minute, $p < 0.05$. In 4 patients with an obstructed anastomosis no statistically significant index changes occurred.

### *9. Patients Complaints*

Before surgery 121 patients complained of pain in the lower extremities. Such pain disappeared postopera-

**Table 1** Anti-Insulin Hormone Level in Blood on Empty Stomach Before (PRE) and After (POST)Surgery.

| *Patient No.* | *Sex* | *Age* | *Duration of disease* | *Insulin dose (U/kg)* | | *Glugagon level (pg/ml)* | | *Cortisol level (nml/l)* | |
|---|---|---|---|---|---|---|---|---|---|
| | | | *years* | *pre* | *post* | *pre* | *post* | *pre* | *post* |
| 1 | M | 19 | 14 | 1.5 | 1.1 | 72.2 | 94.4 | 1911.4 | 1559.9 |
| 2 | M | 38 | 2 | 0.6 | 0.6 | 77.2 | 153.8 | 759.0 | 694.4 |
| 3 | F | 30 | 20 | 0.8 | 0.8 | 21.0 | 76.2 | 827.7 | 388.4 |
| 4 | M | 33 | 7 | 0.5 | 0.14 | 76.1 | 100.5 | 1060.8 | 967.6 |
| 5 | M | 22 | 12 | 0.75 | 0.43 | 60.2 | 86.5 | 1559.4 | 992.6 |
| 6 | M | 32 | 5 | 0.61 | 0.64 | 16.6 | 59.2 | 1436.1 | 1492.2 |
| 7 | F | 24 | 19 | 1.0 | 0.8 | 50.8 | 70.0 | 1850.9 | 779.8 |
| 8 | F | 27 | 19 | 0.98 | 0.77 | 63.3 | 80.4 | 2466.0 | 836.1 |
| 9 | M | 34 | 8 | 1.2 | 1.0 | 68.9 | 196.5 | 1188.9 | 777.5 |

tively in 52 patients with patent anastomoses, abated considerably in 33, and persisted in only 11. At the same time pain persisted in 12 of the 19 patients with obstructed anastomoses, abated in 4 and disappeared in 3 (the difference between the groups by the $x^2$ criterion is statistically significant, $p < 0.001$).

Before surgery hypoglycemia occurred in 110 patients. After surgery 54 patients with patent anastomoses stopped complaining of hypoglycemia, in 27 it occurred seldom, and in only 11 patients the operation produced no improvement. Out of the 18 patients with impassable anastomoses 10 had postoperatively the same complaints, in 6 the hypoglycemia states were more easily tolerated, and in only 2 patients hypoglycemia disappeared (the difference between the groups by the $x^2$ criterion is statistically significant, $p < 0.001$).

### DISCUSSION

Changes in the blood discharged from hormone-producing organs (pancreas and adrenal glands) is used in metabolic surgery to improve the patients' status in glucagon storage disease[10], hereditary hyperlipedemia[11], some forms of hypertension[12], and chronic hepatitis[13]. In these cases the liver, as the chief organ of hormone metabolization, is eitherincluded in[12,13], or excluded from[10,11] the blood flow.

The liver is the chief organ for the metabolism of pancreatic hormones, determining glucose homeostasis. Experiments on diabetes mellitus models show that in the case of disturbances of insulin secretion the liver's glucose production increases and is not blocked by the administration of glucose[14]. One of the causes might be increased glucagon secretion. Also important is insulin degradation in the liver[15], which lowers the content in the systemic blood. On the strength of data about insulin degradation in the liver Le Veen used a splenorenal anastomosis in dogs with subtotal resection of the pancreas in order to save exogenous insulin and observed a drop in the blood glucose level[16]. We have not found reports in the literature confirming such investigations in clinical practice.

In our view, the liver's regulating role in maintaining glucose homeostasis in IDD is limited by the fact that subcutaneously injected insulin is distributed in the main in peripheral tissues, while for endogenous insulin it is the liver. The entry of insulin into the liver while there is relative hypoinsulinemia in the portal vein system and hyperinsulinemia in peripheral tissues creates conditions for noncompensated neoglucogenesis, upsets the formation of glycogen from glucose, complicates the interaction of the liver, muscular and fatty tissues in the exchange of energy substrates (glucose, free fatty acids, lactate and amino acids). This is possibly one of the causative factors in the development of hypo- and hyperglycemic states and determines the difficulty of attaining a stable clinical effect with subcutaneous administration of insulin.

Surgical placement of a distal venous splenorenal anastomosis creates conditions for a more optimal correlation between the injected exogenous insulin and endogenous glucagon in the tissues.

The main point up this communication was to reveal the dependence of surgical results on the patency of the splenorenal anastomosis. Our investigations demonstrated that in an overwhelming majority of patients with a patent anastomosis the main complaints disappeared or considerably abated, and $HbA_{1c}$, an indicator not only of carbohydrate, but also of fat metabolism compensaton was, lowered[17]. In patients with an impassable anastomosis, when there is practically no shunting of glucagon into the systemic blood flow, the complaints persisted, and the $HbA_{1c}$ level did not drop. We excluded the possibility of the surgical results being influenced by the postoperative pattern of insulin therapy. The main factor affecting the results of surgery and the requirement of exogenous insulin, was the patency or impassability of the splenorenal anastomosis.

A number of experiments carried out allow us to presuppose the mechanisms whereby the patients' postoperative improvement takes place. It is well known that disturbances of tissue microcirculation and oxygenation are largely determined by the conditions of microangiopathic development. Epicutaneous measurement in patients with patent anastomoses showed higher oxygen pressures than in cases of impassable anastomoses, whereas this index for arterial blood was the same in both groups. This perhaps points to improvement of capillary blood flow. At the same time the venous blood $pO_2$ and the arterial blood $pCO_2$ in patients with a patent anastomosis was lower than when the anastomosis was obliterated. This gives grounds for noting better oxygen utilization by the tissues after the operation. Improvement of microcirculation may be a factor in the normalization of the GFR and an improvement in the vegetative innervation of the heart.

The examination of glucagon levels yielded important information. Preoperatively the patients' glucagon level in a peripheral vein were practically identical. After surgery a rise of glucagon levels was observed only in patients with patent anastomoses. Thus, an improvement of the clinical status occurred upon an increase of glucagon in the systemic blood stream, which, possibly, created conditions for its more optimal interaction with subcutaneously injected insulin.

There are some things which we are still unable to explain, but which we believe warrant attention. Observed in patients with patent anastomoses was a rise of the c-peptide level on an empty stomach. Whether this results from the improved functioning of the pancreas or is a response to stimulation by endogenous glucagon, which postoperatively affects the beta-cells of the pancreas via the systemic blood flow, is not clear.

Of certain interest is glucagon's interaction with adrenal hormones, which also possess a contrainsular action and play an important role in the regulation of vascular tone and microcirculation. Increased secretion by the adrenal glands of contrainsular hormones in diabetics is regarded as a response to metabolic stress, which during the long term course of diabetes gets transformed into an injury mechanism, promoting the development of microangiopathy[18]. Glucagon and the contrainsular hormones of the adrenals are to a large extent synergists, providing the body with metabolic substrates[1]. At the same time, in distinction to the adrenal hormones, glucagon does not produce a vasospastic effect. As our studies have shown (see Table 1), extrahepatic shunting of glucagon into the systemic bloodflow is accompanied by a diminution of the cortisol level. Further investigations will clarify whether these changes are regular, and establish their role in altering the microcirculation.

An analysis of the findings presented in this paper convincingly points to the dependence of surgical results on the patency of the created splenorenal anastomosis. The chief result of the operation is stabilisation of the diabetic course.

### REFERENCES

1. Roy Taylor and Loranne Agius (1988) The biochemistry of diabetes. Review article. *Biochem. J.* **250**: 625–640.
2. Unger R. H. (1975) Diabetes and the alpha cell. *Diabetes*, **25**: 136–151.
3. Gerich J. E., Lorenzi M., Hane S., Gustafson G., Guillemin R. and Forsham P. H. (1975) Evidence for a physiologic role of pancreatic glucagon in human glucose homeostasis. Studies with somatostatin. *Metabolism*. **24**: 175–182.
4. Unger R. H. and Orci L. (1975) The essential role of glucagon in the pathogenesis of diabetes mellitus. *Lancet*. **1**: 14–16.
5. Galperin E.I., Kuzovlev N.F., Diuzhina T.G. and Alexandrovskaya T.N. (1983). Approach to surgical treatment of diabetes mellitus (Experimental study). *Chirurgia*, **1**: 13–20 (in Russian).
6. Galperin E. I., Diuzheva T. G., Milovanova L. P., Kuzovlev N. F., Gitel E. P. and Alexandrovskaya T. N. (1985). Compensation for insular deficiency by changing the portal circulation. *Bul. exper. biol. med.* **4**: 418–421 (in Russian).
7. Galperin E. I., Shraer T. I., Diuzheva T. G., Milovanova L. P., Kuzovlev N. F., Bolshakova T. D., Rozina N.S. *et al.* (1987) Experimental substantiation and the first clinical experience in surgical; management of diabetes mellitus. *Chirurgia*, **2**: 64–70 (in Russian).
8. Mallia A. K., Hermanson G. T., Krohn R. I., Fujimoto E. K. and Smith P. K. (1981) Preparation and use of a boronic acid affinity support for separation and quantitation of glycosylated hemoglobins. *Anal. Letters*, **14**: 649–660.
9. Clarke B. F., Ewing D. J. and Campbell I. W. (1970). Diabetic autonomic neuropathy. *Diabetologia*, **17**: 195–212.
10. Startzl T.E., Putam S.W. and Porter K.A. (1973) Portal diversion for treatment of glycogen storage disease in humans. *Ann. Surg.*, **176**: 525–539.
11. Startzl T. E., Chase H. P., Putnam C. W. *et al.* (1974) Follow up of patient with portocaval shunt for the treatment of hyperlipidemia, *Lancet*, **2**: 714–715.
12. Pokrovsky A. B., Torgunakov A. P., Kazanchian A. O. and Yaroshchuk A.S. (1983) Placing a portorenal venous anastomosis for the treatment of arterial hypertension, *Chirurgia*, **10**: 99–103 (in Russian).
13. Torgunakov A.P., Krivov Yu. I. and Ponomariov V. N. (1984) New opportunities for the treatment of chronic hepatitis, *Vestnik khirurgii*, **12**: 45–47 (in Russian).
14. Madison L.L. (1969) Role of insulin in the hepatic handling of glucose. *Arch. Intern. Med.*, **123**: 284–292.
15. Mirsky I. A. (1964) The metabolism of insulin, *Diabetes*, **13**: 225–229.
16. Le Veen H. H., Diaz C. A. and Piccone V. A. (1969) A surgical approach to diabetes mellitus, *Amer. J. Surg.*, **117**: 46–54.
17. Grinshpun M.N., Galionok V.A., Mazovetsky A.G. and Dikker V. E. (1988) A comparative analysis of glycolysated hemoglobin determination techniques, *Laboratornoye delo*, **2**:51–54 (in Russian).
18. Yefimov A. S. (1989) Diabetic angiopathies, *Meditsina Publishers. Moscow*, **228**: pp. (in Russian).

